# Identification of direct regulatory targets of the transcription factor Sox10 based on function and conservation

**DOI:** 10.1186/1471-2164-9-408

**Published:** 2008-09-11

**Authors:** Kyung Eun Lee, Seungyoon Nam, Eun-ah Cho, Ikjoo Seong, Jin-Kyung Limb, Sanghyuk Lee, Jaesang Kim

**Affiliations:** 1 Division of Life and Pharmaceutical Sciences and the Center for Cell Signaling & Drug Discovery Research, Ewha Womans University, 11-1 Daehyun-dong, Seodaemun-gu, Seoul, 120-750, Korea; 2 Interdisciplinary Program in Bioinformatics, Seoul National University, San 56-1, Shinlim-dong, Gwanak-gu, Seoul, 151-741, Korea

## Abstract

**Background:**

Sox10, a member of the Sry-related HMG-Box gene family, is a critical transcription factor for several important cell lineages, most notably the neural crest stem cells and the derivative peripheral glial cells and melanocytes. Thus far, only a handful of direct target genes are known for this transcription factor limiting our understanding of the biological network it governs.

**Results:**

We describe identification of multiple direct regulatory target genes of Sox10 through a procedure based on function and conservation. By combining RNA interference technique and DNA microarray technology, we have identified a set of genes that show significant down-regulation upon introduction of Sox10 specific siRNA into Schwannoma cells. Subsequent comparative genomics analyses led to potential binding sites for Sox10 protein conserved across several mammalian species within the genomic region proximal to these genes. Multiple sites belonging to 4 different genes (proteolipid protein, Sox10, extracellular superoxide dismutase, and pleiotrophin) were shown to directly interact with Sox10 by chromatin immunoprecipitation assay. We further confirmed the direct regulation through the identified cis-element for one of the genes, extracellular superoxide dismutase, using electrophoretic mobility shift assay and reporter assay.

**Conclusion:**

In sum, the process of combining differential expression profiling and comparative genomics successfully led to further defining the role of Sox10, a critical transcription factor for the development of peripheral glia. Our strategy utilizing relatively accessible techniques and tools should be applicable to studying the function of other transcription factors.

## Background

Identifying direct targets of a given transcription factor is a key step in dissecting the regulatory network wherein the detailed function of the transcription factor can be understood. Early attempts included ectopic over-expression followed by expression profiling for identification of candidate genes which in turn entailed extensive sequence analyses of the promoter region [1]. Another strategy was direct cloning of DNA fragments associated with transcription factors which also entailed extensive sequence analyses for identification of nearby genes [2].

Several recently developed molecular tools and technical advances have been paving ways for achieving the same goal in a more efficient high throughput manner. For example, microarray expression profiling has dramatically improved the differential screening, and completion of genome sequencing for several species now allows a rapid identification of genes in the genomic context as well as the application of comparative genomics analysis for conserved DNA elements. Furthermore, RNA interference (RNAi) revolutionized somatic cell genetics making it possible to perform loss-of-function experiments conveniently while chromatin immunoprecipitation (ChIP) assay provides a reasonably efficient means of testing direct interaction between proteins and DNA within cellular context. It had been envisioned that these tools, in variable combinations [3,4], can be utilized for identifying direct regulatory targets of transcription factors, and successful cases are being reported [5,6].

Sox10 is an HMG box family transcription factor expressed in neural crest stem cells and a subset of neural crest derived lineages [7-9]. At the stem cell stage, Sox10 plays the role of a stem cell factor maintaining multipotency and inhibiting premature neurogenesis [8]. In glial cells of the peripheral nervous system (PNS) and melanocytes, Sox10 continues to be expressed after their lineage segregation and promotes differentiation by targeting cell type specific genes [10]. The function of Sox10 has been extensively studied in vivo with gene-targeted and spontaneously occurring mutant murine models [7,11,12]. Heterozygous and homozygous loss-of-function mutations of Sox10 result in severe compromises in the development of PNS via glial developmental failure as well as in the proliferation and differentiation of melanocytic lineages. The human patients of Waardenburg-Shah syndrome with mutations in Sox10 show strikingly similar phenotypes to heterozygous mutant murine models typified by megacolon and hypopigmentation [13].

To date, only a handful of direct regulatory targets of Sox10 are known. In the glial lineage, genes such as the P0, myelin basic protein (MBP), ciliary neurotrophic factor (CNTF) and connexin 32 (Cx32) have been shown to be direct targets of Sox10 [14-17]. In melanocytes, Sox10 has been shown to directly regulate at least two genes, microopthalmia (MITF) and dopachrome tautomerase (DCT) [18-20]. It is likely that Sox10 has additional direct target genes in both glial and melanocytic cells during differentiation and in mature stage.

Here, we describe a stream-lined procedure in identifying such direct regulatory targets. We took advantage of RNAi technique to screen for genes in a Schwannoma cell line that are down-regulated upon introducing small interfering RNA (siRNA) specific for Sox10. Genes with significant level of down-regulation were subjected to comparative genomics analysis for identification of potential Sox10 binding sites that are conserved in multiple mammalian species. Next, we confirmed the physical interaction between Sox10 and the predicted target binding sites by chromatin immunoprecipitation (ChIP) assay leading to identification of at least 4 novel direct regulatory targets, proteolipid protein (PLP), Sox10, extracellular superoxide dismutase (SOD3), and pleiotrophin (Ptn). We further confirm the direct regulation of SOD3 by Sox 10 through the identified cis-element using electrophoretic mobility shift assay (EMSA) and reporter assay.

## Results

We have previously reported a case of apparent transdifferentiation of RT4D6 Schwannoma cells into smooth muscle cells upon introduction of an siRNA specific to the transcription factor Sox10 [21]. Accompanying gene expression profiling showed apparent down-regulation of several glial specific marker genes such as GFAP and p75 and up-regulation of smooth muscle specific genes including α-smooth muscle actin confirming the phenomenon of transdifferentiation. Sox10 is known to be a direct transcriptional activator of genes specific to glial lineage including P0 and MBP [16,17]. We reasoned that several of the genes down-regulated by Sox10 specific siRNA are likely to represent additional direct regulatory targets. We further reasoned that such direct regulatory targets have appropriate cis-elements, Sox10 protein binding sites that are conserved across mammalian if not vertebrate species.

Gene expression profiling was carried out with two independent pairs of RT4D6 cell samples each treated with wild type and mutant siRNAs, and 88 affy probes that showed at least 4-fold down-regulation upon introduction of Sox10 specific siRNA were identified (see Methods; additional data file 1). Among these, 44 genes had RefSeq Ids and were subjected to subsequent promoter and comparative genomics analyses (additional data file 1). We examined the genomic region of each gene from -2 kb upstream of the transcription start site to the end of the first intron to identify the conserved potential Sox10 binding sites. Although systematic analyses for preferred binding sites have been carried out for several Sox proteins, no such study has been reported for Sox10 [22-25]. In addition, these sites have been determined through in vitro binding assays which may not fully reflect the in vivo binding preference or potential. We therefore took as the core element the 5 nucleotide sequence, 5'ACAAT3' (or the complementary sequence 5'ATTGT3'), that is targeted by all tested Sox proteins. From the rat genomic sequences examined, profile search using the TRANSFAC database led to the identification of 1328 sites.

**Figure 1:**
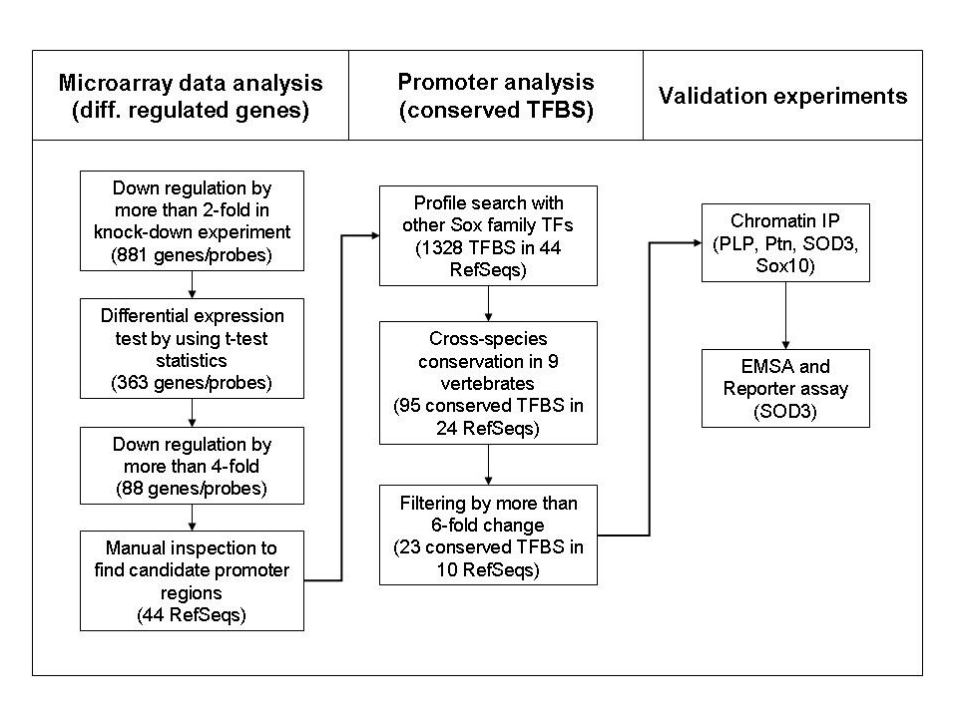
Overview of the study. The study is composed of three main stages. First, the microarray data analysis resulted in 44 significantly down-regulated candidate genes from the siRNA knock down experiment. The second stage process, the promoter analysis, led to the identification of 23 conserved putative Sox10 binding sites belonging to 10 genes. The last stage process includes various wet-lab validation experiments.

**Figure 2:**
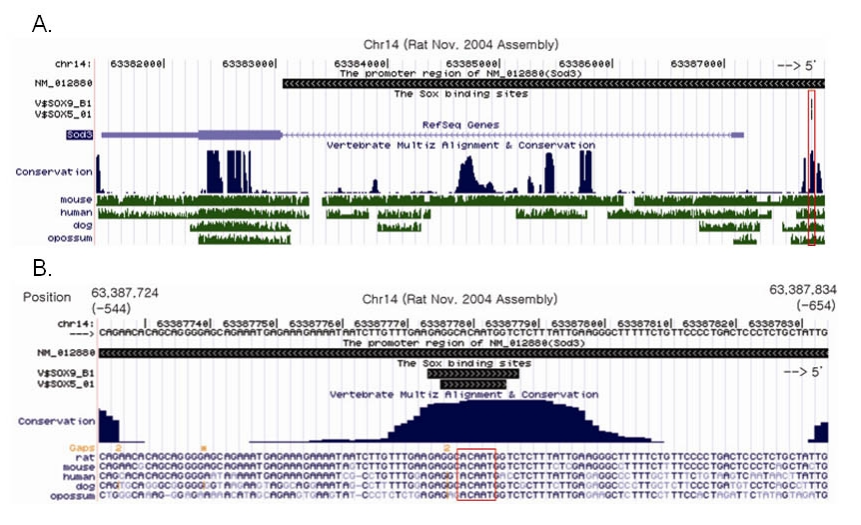
A. SOD3 locus on rat chromosome 14; adapted from the University of California at Santa Cruz (UCSC) Genome Browser. The conserved cluster shown in the Conservation track containing the putative SOX10 TFBS is indicated by the red box near the right end of the panel. The unique site detected by V$SOX9_01 and V$SOX5_01 matrices in the TRANSFAC database is also indicated in the box. B. The nucleotide sequences surrounding conserved binding site is shown in detail. The numbers in the parentheses in the Position track indicate the distance from the SOD3 transcription start site. The red box indicates the conserved core sequence, 5'ACAAT3'.

We next applied the cross-species conservation criteria (see Methods) reasoning that functional Sox10 binding sites are likely conserved across mammalian species. This led to further refinement of putative targets to 95 candidate sites belonging to 24 genes (additional data file 2). For the subsequent validation analyses using 'wet-lab' experimental procedures, further selection was carried out. Specifically, only the genes which showed 6-fold or higher levels of change by specific siRNA and whose core elements were perfectly conserved among all mammalian species with accessible genome were selected. This led to the final 10 genes with 23 putative SOX10 binding sites (table 1). The computational pipeline is summarized in figure 1, and figure 2 shows the gene structure, conserved genomic regions, and nucleotide sequence surrounding the putative binding site of one of the 10 genes, SOD3.

**Table 1:** The candidate 10 genes and 23 putative Sox10 binding sites tested by ChIP assay.

**gene symbol**	**RefSeq ID**	**target cis element**	**chromosome**	**start**	**end**	**Location**	**primer pair #**
Cmkor1	NM_053352	tttATTGTtctc	9	89344237	89344250	Intron	1
		agcACAATgg	9	89346538	89346547	Intron	2
Gda	NM_031776	ttgACAATtt	1	224640748	224640757	Intron	3
PLP	NM_030990	cctATTGTgttt	X	124488088	124488101	5'enhancer	4
		taATTGTttt	X	124492945	124492954	intron	5
		ctATTGTata	X	124493179	124493188	Intron	6
		gaATTGTgtg	X	124493238	124493247	Intron	6
		ggtACAATtg	X	124493249	124493258	Intron	6
		ccATTGTtta	X	124494810	124494819	Intron	7
Sox10	NM_019193	agATTGTcca	7	117150489	117150498	5'enhancer	8
		caATTGTctg	7	117150400	117150409	5'enhancer	8
SOD3	NM_012880	ccATTGTgcc	14	63387776	63387785	5'enhancer	9
Erbb3	NM_017218	tcATTGTggg	7	1874401	1874410	Intron	10
		gacACAATag	7	1874335	1874344	Intron	10
Ptn	NM_017066	caATTGTtgg	4	64147810	64147819	Intron	11
		cacACAATag	4	64147702	64147711	Intron	11
		taATTGTgag	4	64136510	64136519	Intron	12
		atATTGTaac	4	64127690	64127699	Intron	13
		attACAATaa	4	64126376	64126385	Intron	14
Ngfr	NM_012610	tggACAATgg	10	84280255	84280264	Intron	15
		cagACAATgg	10	84280237	84280246	Intron	15
Gzmb	NM_138517	cgATTGTgat	15	35199893	35199902	5'enhancer	16
Gas7	NM_053484	ccATTGTatg	10	54304002	54304011	Intron	17

The number in the last column indicates the primer pair used to test each site by ChIP assay (see additional data file 3).

**Figure 3:**
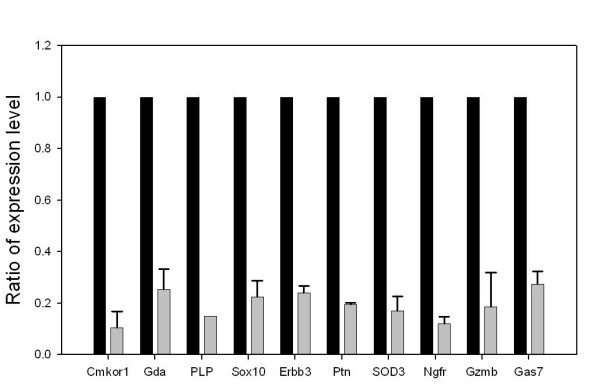
Down-regulation of Sox10 by wild type siRNA results in down-regulation of the 10 target genes. Real time PCR was performed on cDNA preparations from RT4D6 cells transfected with wild type and mutant Sox10 siRNAs. The values shown are the relative ratios of expression level normalized with GAPDH expression level. Data are the average of three independent experiments each with two measurements, and error bars represent standard deviations.

We next sought to confirm the regulatory relationship between Sox10 and the proposed 10 target genes. cDNA preparations were made from RT4D6 cells treated with Sox10 specific and control siRNAs. Real time PCR analyses showed that all the proposed 10 target genes were clearly down-regulated as the result of Sox10 down-regulation confirming the microarray screen results (figure 3).

**Figure 4:**
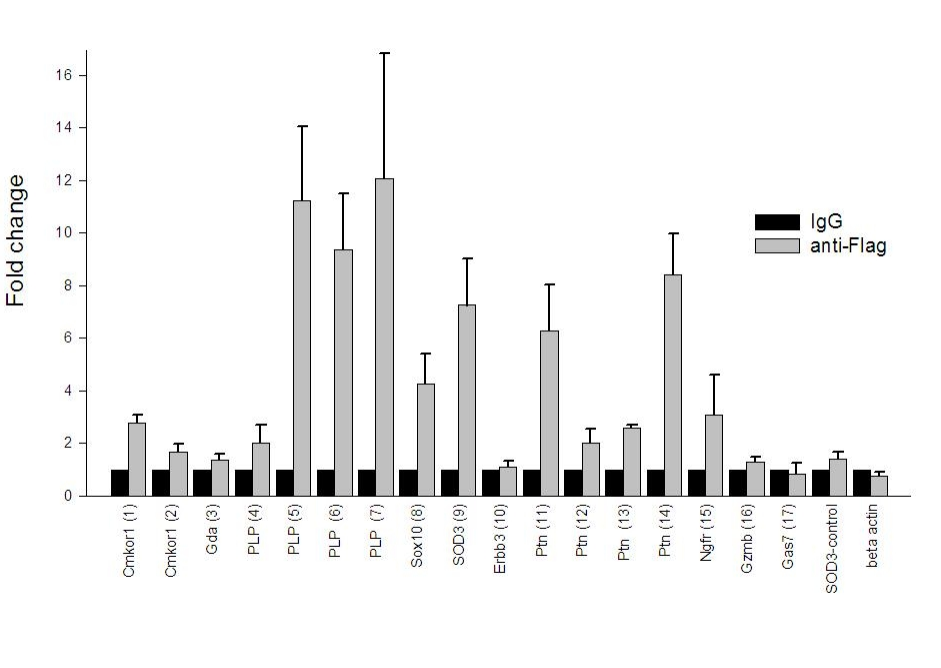
ChIP assay. Chromatin immunoprecipitation was carried out in RT4D6 cells transfected with pFlag-Sox10. Values from immunoprecipitation with anti-Flag antibody represent the fold difference relative to those from IgG control antibody. Number in the parentheses next to the gene name indicates the primer pair used (see table 1 and additional data file 3). Data are the average of three independent experiments each with two measurements, and error bars represent standard deviations.

Chromatin immunoprecipitation assay was performed for the 23 putative Sox10 binding sites (table 1) with RT4D6 cells transfected with a Flag epitope tagged Sox10 construct. Ectopic expression of epitope tagged Sox10 was necessary due to the absence of an antibody that effectively immunoprecipitated the endogenous Sox10 from RT4D6 cells. As some of the potential binding sites were located close to one another, only 17 pairs of primer were used to examine the 23 sites. As a control, each of the sites was examined after precipitation with IgG antibody. We also used β-actin locus and a cis-locus located 10 kb away from the putative Sox10 binding site of SOD3 gene as additional negative controls. The result suggests that at least 4 genes, PLP, Sox10, SOD3, and Ptn contain binding sites that SOX10 interacts with clearly detectable affinity, and Ngfr and Cmkor1 may represent additional Sox10 target genes (figure 4).

We chose SOD3 for further validation as the direct regulatory target of Sox10. The unique candidate binding site, conveniently located in the 5' enhancer region, shows a perfect conservation between rat, mouse, human, dog, and opossum (figure 2).

**Figure 5:**
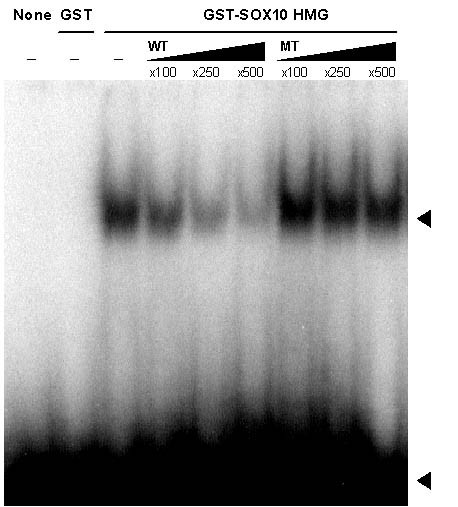
EMSA. EMSA was performed with a ^32^P end-labeled SOD3 probe containing the conserved Sox binding site. Proteins added are noted at the top. The upper arrowhead indicates the GST-SOX10 HMG/DNA probe complex, and the lower arrowhead indicates the free probe. For competition experiments, unlabeled wild type (WT) or mutated (MT) probes were added to the indicated excess levels.

We first confirmed that Sox10 protein can bind to the candidate site by EMSA (figure 5). The DNA binding domain of Sox10 readily formed a protein-DNA complex with a probe spanning the candidate binding element. Importantly, the binding between the protein and the labeled probe was effectively abrogated by specific unlabeled probe but not by non-specific probe which contains mutations in the core SOX binding sequence.

**Figure 6:**
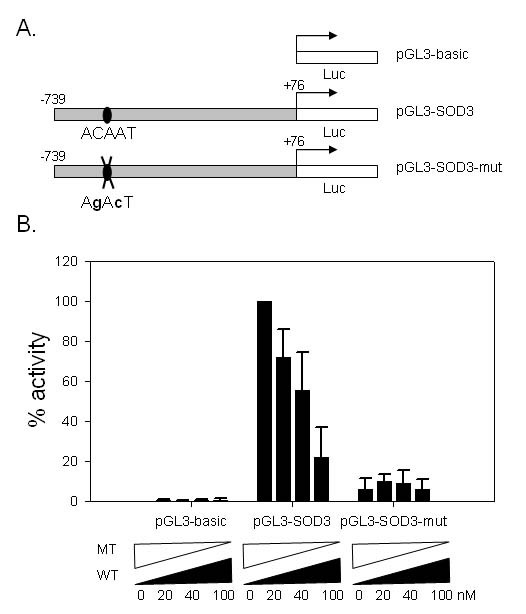
Reporter assay. A. The reporter constructs are illustrated. pGL3-SOD3 contains the wild type SOD3 enhancer sequence while pGL3-SOD3-mut is identical except for the mutations in the core SOX binding element. These are derived from pGL3-basic shown at the top. B. Normalized relative levels of luciferase activity are shown with the value from pGL3-SOD3 in the absence of wild type siRNA as 100%. The combined amount of wild type and mutant siRNAs was equal in all cases. Data are the average of three independent experiments, and error bars represent standard deviations.

Next, we examined the role of the candidate binding site in a reporter assay (figure 6). Two reporter constructs were designed. The wild type SOD3 reporter, pGL3-SOD3, has luciferase gene ligated to the 5' enhancer and UTR region of SOD3 inclusive of the candidate cis-element while mutant promoter pGL3-SOD3-mut is identically designed except for mutations in the core SOX binding element. These reporter plasmids were co-transfected with various combinations of wild type and mutant siRNAs for Sox10. Importantly, at all concentrations of the wild type siRNA, the wild type reporter showed consistently higher activity than the mutant reporter demonstrating the importance of the intact core Sox binding element. The activity of the wild type reporter showed a concentration dependent down-regulation by the wild type siRNA while the mutant reporter showed little variation of the low level activity. Taken together with the results from ChIP assay and EMSA, these data demonstrate that SOD3 is a direct regulatory target of Sox10 which imparts its activity through the conserved Sox binding element in the 5' enhancer region.

## Discussion

We have applied in combination several recently developed techniques and tools of molecular biology to identify multiple novel targets of Sox10. Rather than relying on overexpression of Sox10, we used RNAi to screen for affected genes. Overexpression of transcription factors can potentially activate non-physiological targets or even repress true targets by 'squelching'. Overexpression of transcription factors in cells already expressing the factor may also have minimal or no effects on the level of target gene expression. The loss-of-function approach followed by microarray screen should produce less non-specific side effects and thus lead to more precise profiling of direct and indirect targets. The procedure combining differential expression profiling and comparative genomics analyses represents a reasonably streamlined method accessible for most molecular biology laboratories.

There are clearly more direct target genes for Sox10 than those we have identified here. The number is limited due to several practical reasons. Most importantly, in the end we have examined by 'wet-lab' experiments only the genes that showed at least 6-fold decrease upon down-regulation of Sox10. This excludes the far greater number of genes that are less but meaningfully affected by the change in the level of Sox10. Furthermore, we have restricted our search within the span of -2 kb from the transcription initiation site to the end of the 1^st ^intron. It is well established that regulatory cis-elements often reside outside such span. Lastly, applying less stringent requirements for the core element sequence and the degree of conservation should allow identification of additional candidate targets. It should also be noted that Sox10 plays important roles in other cell types than Schwann cells. These include neural crest stem cells, melanocytes, and oligodendrocytes. Given that Sox10 likely regulates different genes in different cellular contexts, we should expect distinct additional direct targets of Sox10 from these cells. An important merit to our method is that all these limiting parameters can be easily adjusted for expanded search. Obviously, more comprehensive procedures such as ChIP-on-chip or ChIP-Seq screening would produce more direct targets. However, these methods also have inherent limitations and shortcomings and cannot be readily applied to all cases, and developing diverse strategies to identifying targets of transcription factors will continue to be important. An inherent requirement for identifying direct targets of transcription factors is proving through biological functional assays. It should be noted that it is thus difficult to assess the error rates during each of the filtering steps. If and when extensive biological data are collected, a retrospective assessment of the accuracy of prediction from each criterion may be possible. In a sense, our goal in the current study can be described as an application of data exploration techniques to objectively and subjectively identify the genes most likely to yield positive results in our biological assays of binding and transcriptional activation.

We propose four novel direct targets of Sox10. PLP expression has been shown to be rapidly and robustly induced by Sox10 in Tet-inducible Neuro2A cells [26], and PLP is also known to be expressed in Schwann cells [27,28]. Sox10 has been shown to directly regulate other myelin genes including MBP and Cx32 [14,17], and our identification of PLP as another target is consistent with the role that Sox10 plays in peripheral glial cells. Pleiotrophin is a heparin binding secreted soluble factor with multiple functions in various systems [29,30]. Of note it is expressed in glial cells of CNS and PNS during development as well as in adult stages [29,30]. Its up-regulation in Schwann cells after nerve resection also suggest a role in axon regeneration, a process requiring active participation of Schwann cells [31]. SOD3 is the extracellular form of superoxide dismutase that functions as the major extracellular scavenger of superoxide [32]. SOD3 gene-targeted mouse does not show clear phenotypes except for the reduced viability under hyperoxic condition [32]. Nevertheless, under various experimental conditions, the protective role of SOD3 on neural tissues has been shown suggesting that secreted SOD3 may play a role in reducing oxidative stress to neurons and Schwann cells [33,34]. That Sox10 was among the down-regulated genes was not necessarily meaningful given that Sox10 specific siRNA had been applied to the cell. Still, results from the ChIP assay indicate that Sox10 may regulate its own expression. Whether an active positive feedback loop contributes to known functions of Sox10 such as promoting and maintaining glial phenotype should be further explored. While Ngfr (p75) is a well established marker of Schwann cell [8], the expression and function of Cmkor1, chemokine orphan receptor 1 (also known as CXCR7), in Schwann cell lineage have not been investigated thus far. Binding of Sox10 to the putative Sox binding elements belonging to these genes is repeatable, and given the change in the expression level upon down-regulation of Sox10, we would tentatively classify these genes as direct regulatory targets of Sox10. Additional characterization of their binding elements through mutation analyses and reporter assays should further validate the assignment.

## Conclusion

We have developed and successfully applied a streamlined procedure for identification of direct targets of the transcription factor Sox10. The key elements are RNAi based expression profiling and comparative genomics analysis which led to prediction of candidate cis-elements belonging to multiple candidate genes. We subsequently confirmed direct interaction between several of these candidate cis-elements and Sox10 and identified at least 4 different genes as direct regulatory targets of Sox10. Our methodology should be applicable to other transcription factors, and genes thus discovered should be useful in deciphering physiology and function of peripheral glial cells which are critically dependent on Sox10.

## Methods

### Microarray expression profiling

Duplicate expression profiles were obtained with the GeneChip Rat 230 2.0 Array from the Affymetrix Co as has been described [21]. The microarray data have been deposited in the Gene Express Omnibus (GEO) database [GEO:GSE12007]. We subsequently used the GeneSpring GX7.3 for the data analysis. As a pre-filtering step, only the genes classified in "present" or "marginal" categories in both of the duplicate screens with the mutant (control) siRNA were selected in order to include only those genes with detectable level of expression in the absence of inhibition of Sox10. After median normalization, we selected 881 genes that were down-regulated by the wild type (specific) siRNA by more than 2-fold. We next used a t-test statistics to produce 363 differentially expressed genes at the p-value of 0.05. No multiple test correction was applied. Further reduction in the number of candidate genes for subsequent examination was achieved by increasing the threshold to 4-fold reduction which yielded a set of 88 affy probes (additional data file 1). Subsequently, we manually examined the genomic regions specified by the affy probes to select only the genes with RefSeq ID. The 44 genes thus obtained (additional data file 1) were subjected to comparative genomics analysis for the presence of conserved potential Sox10 binding sites.

### Computational identification of the putative Sox binding sites

We developed a computational pipeline that combined the promoter analysis and the comparative genomics analysis. Genomic sequence from the upstream 2 kb point with respect to the transcription start site to the end of the first intron was extracted from the rat genome assembly version 3.4 (Nov. 2004 version in the UCSC genome browser) for the 44 genes. The MATCH program [35] in the TRANSFAC^® ^Professional 10.2 [36] was used to perform the profile search for the transcription factor binding sites (TFBS). The vertebrate binding matrices and the only high quality matrix option were used in the profile selection, and the cut-offs for core and matrix similarity were set to 0.9999 and 0.7 respectively without any change in the other options. The matrix identifiers V$SOX5_01 and V$SOX9_B1 were selected among the several available Sox binding matrices in the TRANSFAC database [22,25]. As the core element, we used 5'ACAAT3' or its complement sequence. Profile search with these two matrices resulted in 1328 putative SOX binding sites which were mapped back to the rat genome assembly. All these procedure were automated by our own browser-emulating Perl program for batch processing.

We next set up a filtering step utilizing the cross-species conservation information. The phastCons score database [37] of 9 species (rat, mouse, human, dog, cow, opossum, chicken, frog, and zebrafish) conservation was downloaded from the UCSC genome browser [38]. The database contains two columns one of which lists genomic coordinates (i.e. base pair position) and the other the corresponding phastCons scores indicating the posterior probability of conservation in a scale of 0 to 1. The conservation score for each TFBS was evaluated to be the average phastCons score within the TFBS region. Setting the conservation cut-off to 0.7, we obtained the final 95 putative Sox binding sites proximal to 24 independent genes (additional data file 2).

For subsequent examination by chromatin immunoprecipitation (ChIP) assay, only the genes with 6-fold or higher level of change from the microarray screen were considered. The identified TFBS were visually inspected for 100% sequence conservation of the core sequence in human, mouse, rat, and dog, and 23 different putative Sox binding sites in 10 genes were finally selected for ChIP assay. The name of the genes and genomic locations for the candidate TFBS are listed in the table 1.

### Recombinant Sox10 constructs for EMSA and ChIP assay

For the production of GST fusion protein, HMG domain of Sox10 (a.a. 80~185) was PCR amplified from a mouse cDNA clone and inserted into pGEX-4T-1 plasmid vector (Amersham). The sequences of primers used were 5'-AGAATTCCTCAGCGGCTACGACTGGACG-3' and 5'-ACTCGAGCTGGGCTGCCTTCCCGTTC-3' [9]. The *E. coli *strain BL21 was transformed with the construct, and protein was purified using glutathione agarose beads after IPTG induction.

pFlag-Sox10 was generated by PCR amplifying the full length Sox10 with a pair of PCR primers, 5'-AGCGGCCGCGCCGAGGAACAAGACCTATC-3' and 5'-AGTCGACCTCTAAGGTCGGGATAGAGTCG-3' and inserting the product into pFLAG-CMV vector (Invitrogen) in frame with the FLAG epitope.

### Chromatin immunoprecipitation (ChIP) assay

For the ChIP assay, 2 μg of pFlag-Sox10 plasmid was transfected into 5 × 10^5 ^RT4D6 cells on a 100 mm dish using Effectene transfection reagent (Qiagen). Typically, cells pooled from 4 plates (2 × 10^6 ^cells) were processed per assay. ChIP assays were performed using EZ ChIP™ Chromatin immunoprecipitation kits (Upstate) following the manufacturer's protocols except for the elution of protein-DNA complex. Specifically, immunoprecipitated DNA-protein complexes were eluted twice by adding 100 μl elution buffer containing 3× FLAG peptide (Sigma; 150 ng/μl of 3× FLAG peptide, 50 mM Tris-HCl, pH 7.4, 150 mM NaCl) for 30 min at 4°C. Detailed protocols are available upon request. For the 23 potential Sox10 binding sites subjected to ChIP assay, only 17 PCR primer pairs were used as cis-elements within 200 bp were examined with the same primer pairs. Primer pairs used for PCR are listed in the additional data file 3.

### Electrophoretic mobility shift assay (EMSA)

A pair of oligonucleotides (5'-GAAGAGGCACAATGGTCTCT-3'; 5'-ACCATTGTGCCTCTTC-3') encompassing the candidate SOX10 binding sequence (-590 to -610 5' to the transcription initiation site of SOD3) were hybridized and ^32^P end-labeled for use as the probe. Unlabeled oligonucleotide probe and core sequence mutated oligonucleotide probe (5'-GAAGAGGCAGACTGGTCTCT-3'; 5'-AGAGACCAGTCTGCCTCTTC-3') were used as the specific and non-specific competitors respectively. ^32^P end-labeled probe was incubated with GST-SOX10 HMG protein for 25 min at room temperature in 35 μl of reaction mixture [10 mM HEPES (pH 8.0), 5% glycerol, 50 mM NaCl, 5 mM MgCl_2_, 2 mM DTT, 0.1 mM EDTA, 4 μg of bovine serum albumin, and 2 μg of poly(dIdC)]. For competition experiments, GST-SOX10 HMG protein was preincubated with specified amount of unlabeled probe for 15 min before exposure to the labeled specific probe. The protein-DNA complexes were resolved by 5% polyacrylamide gel electrophoresis in 1× TBE buffer.

### Real Time PCR

Fluorescence real time PCR was performed with SYBR Green PCR master mix (Applied Biosystems) following the manufacturer's protocol. Oligonucleotide primers used as part of the ChIP assay are listed in additional data file 3, and primers used to confirm the microarray screen data are listed in additional data file 4. Preparation of cDNA from siRNA treated RT4D6 Schwannoma cells has been described [21].

### Reporter assay

The promoter region of SOD3 (-739 to +76) was PCR amplified with a pair of primers (5'-ACAGGACAGACCCAGCTAGAG-3', 5'-ACCACAGTCCTGGAGAGAGTG-3') using the BAC clone CH230-303F11 (Children's Hospital Oakland Research Institute) as the template. The resulting DNA fragment was inserted into the 5' region of pGL3 basic promoter (Promega) to produce the wild type reporter construct. The reporter construct with mutated Sox10 binding site was generated using QuickChangeII Site-directed Mutagenesis Kit and a pair of mutagenizing oligonucleotide primers (5'-GTTTGAAGAGGCAGACTGGTCTCTTTATTG-3', 5'-CAATAAAGAGACCAGTCTGCCTCTTCAAAC-3'). For reporter assays, 3 × 10^4 ^RT4D6 cells were seeded on 12 well plates 24 hours prior to transfection. SOD3 reporter plasmids (300 ng), pCMV β-gal plasmids (50 ng), and specified combinations of wild type and mutant siRNAs (combined concentration of 100 nM) were transfected using Lipofectamine 2000 (Invitrogen). After 24 hours, cells were harvested and luciferase activity was measured and normalized using Luciferase Assay System (Promega)

## Authors' contributions

KL carried out the ChIP assay and contributed to preparing the manuscript. SN carried out bioinformatics analyses. EC carried out the RTPCR and reporter assay. IS carried out the EMSA. JL organized the data and contributed to preparing the manuscript. SL designed the bioinformatics analyses and drafted the manuscript. JK designed the 'wet-lab' experiments and drafted the manuscript.

## Supplementary Material

Additional file 1 Genes down-regulated by Sox10 specific siRNA. The 88 probe sets/genes that show more than 4 fold down-regulation by wild type Sox10 siRNA. 44 genes in bold letters with RefSeq ID were subsequently subjected to comparative genomics analysis. Click here for file

Additional file 2 Candidate Sox10 protein binding sites. The 95 conserved putative SOX10 binding sites. The location of each site on rat chromosome is indicated and linked to UCSC genome browser. Click here for file

Additional file 3 Oligonucleotide primers used for ChIP assay. List and sequence of oligonucleotide primers used for ChIP assay. Click here for file

Additional file 4 Oligonucleotide primers used for RT-PCR. List and sequence of oligonucleotide primers used for RT-PCRassay. Click here for file
