# Supplementary material for: Identification of direct regulatory targets of the transcription factor Sox10 based on function and conservation
Source: BMC Genomics. 2008 Sep 11;9:408. doi: 10.1186/1471-2164-9-408 (PMC2556353; doi:10.1186/1471-2164-9-408)
Supplement: Additional file 1 — Genes down-regulated by Sox10 specific siRNA. The 88 probe sets/genes that show more than 4 fold down-regulation by wild type Sox10 siRNA. 44 genes in bold letters with RefSeq ID were subsequently subjected to comparative genomics analysis. [file 1471-2164-9-408-S1.doc]

| **Gene Name** | **Fold Change** | **Genbank** | **Gene Symbol** | **Corresponding RefSeq** |
| --- | --- | --- | --- | --- |
| **1371126_x_at** | **58.85** | **X66693** | **Gzmb^†^** | **NM_138517** |
| 1373336_at | 50.25 | AI411074 |  |  |
| **1368148_at** | **26.63** | **NM_012610** | **Ngfr** | **NM_012610** |
| **1387112_at** | **25.5** | **NM_030990** | **Plp** | **NM_030990** |
| 1398464_at | 22.62 | AI575255 |  |  |
| 1382227_at | 14.98 | AW533203 |  |  |
| 1368513_at | 14.38 | AW144216 | Enpep |  |
| **1370628_at** | **13.98** | **M34097** | **Gzmb^†^** | **NM_138517** |
| **1374540_at** | **13.92** | **AA859235** | **Cdca7_predicted** | **NM_001025693** |
| **1369320_at** | **12.58** | **NM_030852** | **Mia** | **NM_030852** |
| **1379794_at** | **11.9** | **AI029386** | **Gzmb^†^** | **NM_138517** |
| **1368439_at** | **11.55** | **NM_019193** | **Sox10** | **NM_019193** |
| **1373654_at** | **10.41** | **BM389254** | **Anxa8_predicted** | **NM_001031654** |
| 1371485_at | 10.22 | BI283695 |  |  |
| **1369117_at** | **10.09** | **NM_017338** | **Calca^†^** | **NM_017338 ; NM_001033955** |
| 1385956_at | 9.724 | BF289086 |  |  |
| **1369968_at** | **9.4** | **NM_017066** | **Ptn** | **NM_017066** |
| **1368121_at** | **8.722** | **NM_013215** | **Akr7a3** | **NM_013215** |
| **1367940_at** | **8.365** | **NM_053352** | **Cmkor1** | **NM_053352** |
| **1370963_at** | **7.966** | **AJ131902** | **Gas7** | **NM_053484** |
| **1377821_at** | **7.873** | **BI284288** | **Erbb3** | **NM_017218** |
| 1382431_at | 7.635 | AI103530 | Abca1 |  |
| **1370414_at** | **7.407** | **M94043** | **Rab38** | **NM_145774** |
| **1386903_at** | **7.385** | **NM_013191** | **S100b** | **NM_013191** |
| 1370312_at | 7.266 | M88469 | Spon1 |  |
| 1377631_at | 7.111 | BM389001 | Col9a3_predicted |  |
| **1370775_a_at** | **6.864** | **M11597** | **Calca^†^** | **NM_017338 ; NM_001033955** |
| **1387659_at** | **6.72** | **AF245172** | **Gda** | **NM_031776** |
| 1373807_at | 6.64 | AI175732 | Vegf |  |
| 1371491_at | 6.63 | BM390614 | Notch1**^†^** |  |
| **1369370_s_at** | **6.607** | **NM_023970** | **Trpv4 ; Trpv1** | **NM_023970 ; NM_031982** |
| **1369116_a_at** | **6.584** | **NM_017338** | **Calca^†^** | **NM_017338 ; NM_001033955** |
| **1368322_at** | **6.502** | **NM_012880** | **Sod3** | **NM_012880** |
| 1396072_at | 6.234 | BG662585 |  |  |
| 1382358_at | 6.181 | AA943771 | Abcc9 |  |
| 1384479_at | 6.166 | BM386119 |  |  |
| 1368174_at | 6.078 | NM_019371 | LOC497816 |  |
| 1383702_at | 5.901 | AW534301 |  |  |
| **1370068_at** | **5.811** | **NM_017174** | **Pla2g5** | **NM_017174** |
| 1398582_at | 5.799 | AI045780 |  |  |
| **1388918_at** | **5.566** | **BG378074** | **Hdlbp** | **NM_172039** |
| 1372763_at | 5.531 | AI407545 |  |  |
| 1375531_at | 5.472 | BI300956 | LOC299830 |  |
| 1390426_at | 5.429 | BF389398 | Notch1**^†^** |  |
| **1386908_at** | **5.347** | **NM_022278** | **Glrx1** | **NM_022278** |
| **1376800_at** | **5.323** | **AA892496** | **Chn2** | **NM_032084** |
| 1395333_at | 5.281 | AW533483 |  |  |
| 1372091_at | 5.266 | BI275959 | RGD:1303258 |  |
| **1368353_at** | **5.243** | **NM_017009** | **Gfap** | **NM_017009** |
| **1370334_at** | **5.238** | **AF081582** | **Plekhb1** | **NM_172033** |
| 1376038_at | 5.159 | AI411054 | Tex2_predicted |  |
| 1369213_at | 5.152 | NM_017345 | RGD:619777 |  |
| **1374178_at** | **5.005** | **AI103954** | **Arl5** | **NM_053979** |
| 1370942_at | 4.986 | AI170661 | Rasa3 |  |
| 1377336_at | 4.938 | BI275485 | Sema3b_predicted |  |
| 1381995_at | 4.93 | AW530502 | Brunol4_predicted |  |
| **1369167_at** | **4.825** | **NM_012750** | **Gfra2** | **NM_012750** |
| **1381490_at** | **4.814** | **AI711004** | **Phkg1** | **NM_031573** |
| 1371186_at | 4.793 | AA955091 | Itga6 |  |
| **1376263_at** | **4.78** | **BG378672** | **RGD1306222_predicted** | **NM_001009962** |
| **1393649_at** | **4.741** | **BE108758** | **Tmpo** | **NM_012887** |
| **1368484_at** | **4.708** | **NM_022238** | **Abcb9** | **NM_022238** |
| **1390818_at** | **4.703** | **BF288088** | **Atp1b3** | **NM_012913** |
| **1385212_at** | **4.626** | **AI603192** | **LOC302898** | **NM_001008876** |
| **1387169_at** | **4.614** | **NM_053400** | **Tle3** | **NM_053400** |
| 1373019_at | 4.559 | AI172468 | Eps15 |  |
| 1377307_at | 4.535 | BM385387 | LOC498968 |  |
| **1393563_at** | **4.471** | **BF391914** | **Il1rap** | **NM_012968** |
| 1376649_at | 4.444 | AI111965 |  |  |
| 1381635_at | 4.442 | BF404357 | Col18a1 |  |
| **1370245_at** | **4.439** | **AI232474** | **Ctsl** | **NM_013156** |
| 1371588_at | 4.437 | AA686007 | Parva |  |
| 1377232_at | 4.419 | BF406608 | RGD1305269_predicted |  |
| 1377356_at | 4.321 | AA963795 | LOC499211 |  |
| 1398389_at | 4.309 | AI236136 | Pafah1b1 |  |
| **1382967_at** | **4.291** | **BF388060** | **Gpr64** | **NM_181366** |
| **1387106_at** | **4.284** | **NM_022693** | **Sh3bp4** | **NM_022693** |
| **1372101_at** | **4.279** | **AI177031** | **Ppap2b** | **NM_138905** |
| 1388972_at | 4.274 | BF386649 | Rtn4r |  |
| 1374902_at | 4.26 | AI071398 | Iqgap3_predicted |  |
| **1387772_at** | **4.191** | **NM_031969** | **Calm1** | **NM_031969** |
| **1383408_at** | **4.171** | **BI276349** | **Ube2g1** | **NM_022690** |
| **1369443_at** | **4.156** | **NM_133569** | **Angptl2** | **NM_133569** |
| 1372728_at | 4.154 | BE103745 | Sort1 |  |
| **1387897_at** | **4.146** | **L16532** | **Cnp1** | **NM_012809** |
| 1372110_at | 4.132 | BE113148 |  |  |
| 1375073_at | 4.088 | BE107313 | LOC500601 |  |
| 1384148_at | 4.037 | BI293985 |  |  |
| **^†^** : Genes identified by multiple probe sets. | | | | |
