# Supplementary material for: Identification of direct regulatory targets of the transcription factor Sox10 based on function and conservation
Source: BMC Genomics. 2008 Sep 11;9:408. doi: 10.1186/1471-2164-9-408 (PMC2556353; doi:10.1186/1471-2164-9-408)
Supplement: Additional file 2 — Candidate Sox10 protein binding sites. The 95 conserved putative SOX10 binding sites. The location of each site on rat chromosome is indicated and linked to UCSC genome browser. [file 1471-2164-9-408-S2.doc]

| **chromosome** | **start** | **end** | **RefSeqID** | **link** |
| --- | --- | --- | --- | --- |
| chrX | 55659796 | 55659800 | NM_181366 | [Gpr64](http://genome.ucsc.edu/cgi-bin/hgTracks?org=Rat&db=rn4&position=chrX:55659796-55659800) |
| chrX | 55655677 | 55655681 | NM_181366 | [Gpr64](http://genome.ucsc.edu/cgi-bin/hgTracks?org=Rat&db=rn4&position=chrX:55655677-55655681) |
| chr8 | 65485030 | 65485034 | NM_053400 | [Tle3](http://genome.ucsc.edu/cgi-bin/hgTracks?org=Rat&db=rn4&position=chr8:65485030-65485034) |
| chr8 | 65485297 | 65485301 | NM_053400 | [Tle3](http://genome.ucsc.edu/cgi-bin/hgTracks?org=Rat&db=rn4&position=chr8:65485297-65485301) |
| chr9 | 89344241 | 89344245 | NM_053352 | [Cmkor1](http://genome.ucsc.edu/cgi-bin/hgTracks?org=Rat&db=rn4&position=chr9:89344241-89344245) |
| chr9 | 89346541 | 89346545 | NM_053352 | [Cmkor1](http://genome.ucsc.edu/cgi-bin/hgTracks?org=Rat&db=rn4&position=chr9:89346541-89346545) |
| chr6 | 124476411 | 124476415 | NM_031969 | [Calm1](http://genome.ucsc.edu/cgi-bin/hgTracks?org=Rat&db=rn4&position=chr6:124476411-124476415) |
| chr1 | 224656333 | 224656337 | NM_031776 | [Gda](http://genome.ucsc.edu/cgi-bin/hgTracks?org=Rat&db=rn4&position=chr1:224656333-224656337) |
| chr1 | 224640750 | 224640754 | NM_031776 | [Gda](http://genome.ucsc.edu/cgi-bin/hgTracks?org=Rat&db=rn4&position=chr1:224640750-224640754) |
| chr1 | 224618760 | 224618764 | NM_031776 | [Gda](http://genome.ucsc.edu/cgi-bin/hgTracks?org=Rat&db=rn4&position=chr1:224618760-224618764) |
| chrX | 124488092 | 124488096 | NM_030990 | [Plp](http://genome.ucsc.edu/cgi-bin/hgTracks?org=Rat&db=rn4&position=chrX:124488092-124488096) |
| chrX | 124492947 | 124492951 | NM_030990 | [Plp](http://genome.ucsc.edu/cgi-bin/hgTracks?org=Rat&db=rn4&position=chrX:124492947-124492951) |
| chrX | 124493181 | 124493185 | NM_030990 | [Plp](http://genome.ucsc.edu/cgi-bin/hgTracks?org=Rat&db=rn4&position=chrX:124493181-124493185) |
| chrX | 124493240 | 124493244 | NM_030990 | [Plp](http://genome.ucsc.edu/cgi-bin/hgTracks?org=Rat&db=rn4&position=chrX:124493240-124493244) |
| chrX | 124493252 | 124493256 | NM_030990 | [Plp](http://genome.ucsc.edu/cgi-bin/hgTracks?org=Rat&db=rn4&position=chrX:124493252-124493256) |
| chrX | 124494812 | 124494816 | NM_030990 | [Plp](http://genome.ucsc.edu/cgi-bin/hgTracks?org=Rat&db=rn4&position=chrX:124494812-124494816) |
| chr9 | 92732055 | 92732059 | NM_172039 | [Hdlbp](http://genome.ucsc.edu/cgi-bin/hgTracks?org=Rat&db=rn4&position=chr9:92732055-92732059) |
| chr9 | 92726714 | 92726718 | NM_172039 | [Hdlbp](http://genome.ucsc.edu/cgi-bin/hgTracks?org=Rat&db=rn4&position=chr9:92726714-92726718) |
| chr9 | 92726703 | 92726707 | NM_172039 | [Hdlbp](http://genome.ucsc.edu/cgi-bin/hgTracks?org=Rat&db=rn4&position=chr9:92726703-92726707) |
| chr10 | 59495134 | 59495138 | NM_022690 | [Ube2g1](http://genome.ucsc.edu/cgi-bin/hgTracks?org=Rat&db=rn4&position=chr10:59495134-59495138) |
| chr10 | 59496614 | 59496618 | NM_022690 | [Ube2g1](http://genome.ucsc.edu/cgi-bin/hgTracks?org=Rat&db=rn4&position=chr10:59496614-59496618) |
| chr10 | 59496767 | 59496771 | NM_022690 | [Ube2g1](http://genome.ucsc.edu/cgi-bin/hgTracks?org=Rat&db=rn4&position=chr10:59496767-59496771) |
| chr10 | 59497036 | 59497040 | NM_022690 | [Ube2g1](http://genome.ucsc.edu/cgi-bin/hgTracks?org=Rat&db=rn4&position=chr10:59497036-59497040) |
| chr10 | 59500569 | 59500573 | NM_022690 | [Ube2g1](http://genome.ucsc.edu/cgi-bin/hgTracks?org=Rat&db=rn4&position=chr10:59500569-59500573) |
| chr10 | 59500867 | 59500871 | NM_022690 | [Ube2g1](http://genome.ucsc.edu/cgi-bin/hgTracks?org=Rat&db=rn4&position=chr10:59500867-59500871) |
| chr10 | 59505443 | 59505447 | NM_022690 | [Ube2g1](http://genome.ucsc.edu/cgi-bin/hgTracks?org=Rat&db=rn4&position=chr10:59505443-59505447) |
| chr10 | 59512358 | 59512362 | NM_022690 | [Ube2g1](http://genome.ucsc.edu/cgi-bin/hgTracks?org=Rat&db=rn4&position=chr10:59512358-59512362) |
| chr10 | 59521371 | 59521375 | NM_022690 | [Ube2g1](http://genome.ucsc.edu/cgi-bin/hgTracks?org=Rat&db=rn4&position=chr10:59521371-59521375) |
| chr10 | 59529751 | 59529755 | NM_022690 | [Ube2g1](http://genome.ucsc.edu/cgi-bin/hgTracks?org=Rat&db=rn4&position=chr10:59529751-59529755) |
| chr10 | 59537665 | 59537669 | NM_022690 | [Ube2g1](http://genome.ucsc.edu/cgi-bin/hgTracks?org=Rat&db=rn4&position=chr10:59537665-59537669) |
| chr10 | 59545282 | 59545286 | NM_022690 | [Ube2g1](http://genome.ucsc.edu/cgi-bin/hgTracks?org=Rat&db=rn4&position=chr10:59545282-59545286) |
| chr10 | 59548461 | 59548465 | NM_022690 | [Ube2g1](http://genome.ucsc.edu/cgi-bin/hgTracks?org=Rat&db=rn4&position=chr10:59548461-59548465) |
| chr7 | 117150492 | 117150496 | NM_019193 | [Sox10](http://genome.ucsc.edu/cgi-bin/hgTracks?org=Rat&db=rn4&position=chr7:117150492-117150496) |
| chr7 | 117150403 | 117150407 | NM_019193 | [Sox10](http://genome.ucsc.edu/cgi-bin/hgTracks?org=Rat&db=rn4&position=chr7:117150403-117150407) |
| chr7 | 1874404 | 1874408 | NM_017218 | [Erbb3](http://genome.ucsc.edu/cgi-bin/hgTracks?org=Rat&db=rn4&position=chr7:1874404-1874408) |
| chr7 | 1874337 | 1874341 | NM_017218 | [Erbb3](http://genome.ucsc.edu/cgi-bin/hgTracks?org=Rat&db=rn4&position=chr7:1874337-1874341) |
| chr4 | 64158104 | 64158108 | NM_017066 | [Ptn](http://genome.ucsc.edu/cgi-bin/hgTracks?org=Rat&db=rn4&position=chr4:64158104-64158108) |
| chr4 | 64157974 | 64157978 | NM_017066 | [Ptn](http://genome.ucsc.edu/cgi-bin/hgTracks?org=Rat&db=rn4&position=chr4:64157974-64157978) |
| chr4 | 64147813 | 64147817 | NM_017066 | [Ptn](http://genome.ucsc.edu/cgi-bin/hgTracks?org=Rat&db=rn4&position=chr4:64147813-64147817) |
| chr4 | 64147776 | 64147780 | NM_017066 | [Ptn](http://genome.ucsc.edu/cgi-bin/hgTracks?org=Rat&db=rn4&position=chr4:64147776-64147780) |
| chr4 | 64147704 | 64147708 | NM_017066 | [Ptn](http://genome.ucsc.edu/cgi-bin/hgTracks?org=Rat&db=rn4&position=chr4:64147704-64147708) |
| chr4 | 64136513 | 64136517 | NM_017066 | [Ptn](http://genome.ucsc.edu/cgi-bin/hgTracks?org=Rat&db=rn4&position=chr4:64136513-64136517) |
| chr4 | 64129868 | 64129872 | NM_017066 | [Ptn](http://genome.ucsc.edu/cgi-bin/hgTracks?org=Rat&db=rn4&position=chr4:64129868-64129872) |
| chr4 | 64127693 | 64127697 | NM_017066 | [Ptn](http://genome.ucsc.edu/cgi-bin/hgTracks?org=Rat&db=rn4&position=chr4:64127693-64127697) |
| chr4 | 64126441 | 64126445 | NM_017066 | [Ptn](http://genome.ucsc.edu/cgi-bin/hgTracks?org=Rat&db=rn4&position=chr4:64126441-64126445) |
| chr4 | 64126378 | 64126382 | NM_017066 | [Ptn](http://genome.ucsc.edu/cgi-bin/hgTracks?org=Rat&db=rn4&position=chr4:64126378-64126382) |
| chr4 | 64126369 | 64126373 | NM_017066 | [Ptn](http://genome.ucsc.edu/cgi-bin/hgTracks?org=Rat&db=rn4&position=chr4:64126369-64126373) |
| chr10 | 92068132 | 92068136 | NM_017009 | [Gfap](http://genome.ucsc.edu/cgi-bin/hgTracks?org=Rat&db=rn4&position=chr10:92068132-92068136) |
| chr20 | 12806077 | 12806081 | NM_013191 | [S100b](http://genome.ucsc.edu/cgi-bin/hgTracks?org=Rat&db=rn4&position=chr20:12806077-12806081) |
| chr11 | 76218217 | 76218221 | NM_012968 | [Il1rap](http://genome.ucsc.edu/cgi-bin/hgTracks?org=Rat&db=rn4&position=chr11:76218217-76218221) |
| chr11 | 76215859 | 76215863 | NM_012968 | [Il1rap](http://genome.ucsc.edu/cgi-bin/hgTracks?org=Rat&db=rn4&position=chr11:76215859-76215863) |
| chr11 | 76215846 | 76215850 | NM_012968 | [Il1rap](http://genome.ucsc.edu/cgi-bin/hgTracks?org=Rat&db=rn4&position=chr11:76215846-76215850) |
| chr11 | 76214028 | 76214032 | NM_012968 | [Il1rap](http://genome.ucsc.edu/cgi-bin/hgTracks?org=Rat&db=rn4&position=chr11:76214028-76214032) |
| chr11 | 76194304 | 76194308 | NM_012968 | [Il1rap](http://genome.ucsc.edu/cgi-bin/hgTracks?org=Rat&db=rn4&position=chr11:76194304-76194308) |
| chr11 | 76194270 | 76194274 | NM_012968 | [Il1rap](http://genome.ucsc.edu/cgi-bin/hgTracks?org=Rat&db=rn4&position=chr11:76194270-76194274) |
| chr11 | 76183213 | 76183217 | NM_012968 | [Il1rap](http://genome.ucsc.edu/cgi-bin/hgTracks?org=Rat&db=rn4&position=chr11:76183213-76183217) |
| chr8 | 101433078 | 101433082 | NM_012913 | [Atp1b3](http://genome.ucsc.edu/cgi-bin/hgTracks?org=Rat&db=rn4&position=chr8:101433078-101433082) |
| chr8 | 101419871 | 101419875 | NM_012913 | [Atp1b3](http://genome.ucsc.edu/cgi-bin/hgTracks?org=Rat&db=rn4&position=chr8:101419871-101419875) |
| chr14 | 63387779 | 63387783 | NM_012880 | [Sod3](http://genome.ucsc.edu/cgi-bin/hgTracks?org=Rat&db=rn4&position=chr14:63387779-63387783) |
| chr10 | 89520124 | 89520128 | NM_012809 | [Cnp1](http://genome.ucsc.edu/cgi-bin/hgTracks?org=Rat&db=rn4&position=chr10:89520124-89520128) |
| chr15 | 51277842 | 51277846 | NM_012750 | [Gfra2](http://genome.ucsc.edu/cgi-bin/hgTracks?org=Rat&db=rn4&position=chr15:51277842-51277846) |
| chr10 | 84282990 | 84282994 | NM_012610 | [Ngfr](http://genome.ucsc.edu/cgi-bin/hgTracks?org=Rat&db=rn4&position=chr10:84282990-84282994) |
| chr10 | 84280257 | 84280261 | NM_012610 | [Ngfr](http://genome.ucsc.edu/cgi-bin/hgTracks?org=Rat&db=rn4&position=chr10:84280257-84280261) |
| chr10 | 84280239 | 84280243 | NM_012610 | [Ngfr](http://genome.ucsc.edu/cgi-bin/hgTracks?org=Rat&db=rn4&position=chr10:84280239-84280243) |
| chr10 | 84275503 | 84275507 | NM_012610 | [Ngfr](http://genome.ucsc.edu/cgi-bin/hgTracks?org=Rat&db=rn4&position=chr10:84275503-84275507) |
| chr10 | 4283240 | 4283244 | NM_001008876 | [LOC302898](http://genome.ucsc.edu/cgi-bin/hgTracks?org=Rat&db=rn4&position=chr10:4283240-4283244) |
| chr10 | 4283415 | 4283419 | NM_001008876 | [LOC302898](http://genome.ucsc.edu/cgi-bin/hgTracks?org=Rat&db=rn4&position=chr10:4283415-4283419) |
| chr10 | 4283663 | 4283667 | NM_001008876 | [LOC302898](http://genome.ucsc.edu/cgi-bin/hgTracks?org=Rat&db=rn4&position=chr10:4283663-4283667) |
| chr10 | 4283869 | 4283873 | NM_001008876 | [LOC302898](http://genome.ucsc.edu/cgi-bin/hgTracks?org=Rat&db=rn4&position=chr10:4283869-4283873) |
| chr10 | 4285665 | 4285669 | NM_001008876 | [LOC302898](http://genome.ucsc.edu/cgi-bin/hgTracks?org=Rat&db=rn4&position=chr10:4285665-4285669) |
| chr10 | 4286886 | 4286890 | NM_001008876 | [LOC302898](http://genome.ucsc.edu/cgi-bin/hgTracks?org=Rat&db=rn4&position=chr10:4286886-4286890) |
| chr10 | 4287661 | 4287665 | NM_001008876 | [LOC302898](http://genome.ucsc.edu/cgi-bin/hgTracks?org=Rat&db=rn4&position=chr10:4287661-4287665) |
| chr5 | 126123477 | 126123481 | NM_138905 | [Ppap2b](http://genome.ucsc.edu/cgi-bin/hgTracks?org=Rat&db=rn4&position=chr5:126123477-126123481) |
| chr5 | 126129128 | 126129132 | NM_138905 | [Ppap2b](http://genome.ucsc.edu/cgi-bin/hgTracks?org=Rat&db=rn4&position=chr5:126129128-126129132) |
| chr5 | 126133137 | 126133141 | NM_138905 | [Ppap2b](http://genome.ucsc.edu/cgi-bin/hgTracks?org=Rat&db=rn4&position=chr5:126133137-126133141) |
| chr5 | 126134974 | 126134978 | NM_138905 | [Ppap2b](http://genome.ucsc.edu/cgi-bin/hgTracks?org=Rat&db=rn4&position=chr5:126134974-126134978) |
| chr5 | 126146458 | 126146462 | NM_138905 | [Ppap2b](http://genome.ucsc.edu/cgi-bin/hgTracks?org=Rat&db=rn4&position=chr5:126146458-126146462) |
| chr5 | 126148823 | 126148827 | NM_138905 | [Ppap2b](http://genome.ucsc.edu/cgi-bin/hgTracks?org=Rat&db=rn4&position=chr5:126148823-126148827) |
| chr5 | 126152996 | 126153000 | NM_138905 | [Ppap2b](http://genome.ucsc.edu/cgi-bin/hgTracks?org=Rat&db=rn4&position=chr5:126152996-126153000) |
| chr5 | 126154372 | 126154376 | NM_138905 | [Ppap2b](http://genome.ucsc.edu/cgi-bin/hgTracks?org=Rat&db=rn4&position=chr5:126154372-126154376) |
| chr5 | 126156780 | 126156784 | NM_138905 | [Ppap2b](http://genome.ucsc.edu/cgi-bin/hgTracks?org=Rat&db=rn4&position=chr5:126156780-126156784) |
| chr15 | 35199921 | 35199925 | NM_138517 | [Gzmb](http://genome.ucsc.edu/cgi-bin/hgTracks?org=Rat&db=rn4&position=chr15:35199921-35199925) |
| chr15 | 35199903 | 35199907 | NM_138517 | [Gzmb](http://genome.ucsc.edu/cgi-bin/hgTracks?org=Rat&db=rn4&position=chr15:35199903-35199907) |
| chr15 | 35199896 | 35199900 | NM_138517 | [Gzmb](http://genome.ucsc.edu/cgi-bin/hgTracks?org=Rat&db=rn4&position=chr15:35199896-35199900) |
| chr3 | 12228282 | 12228286 | NM_133569 | [Angptl2](http://genome.ucsc.edu/cgi-bin/hgTracks?org=Rat&db=rn4&position=chr3:12228282-12228286) |
| chr3 | 12230177 | 12230181 | NM_133569 | [Angptl2](http://genome.ucsc.edu/cgi-bin/hgTracks?org=Rat&db=rn4&position=chr3:12230177-12230181) |
| chr3 | 12234190 | 12234194 | NM_133569 | [Angptl2](http://genome.ucsc.edu/cgi-bin/hgTracks?org=Rat&db=rn4&position=chr3:12234190-12234194) |
| chr10 | 54304004 | 54304008 | NM_053484 | [Gas7](http://genome.ucsc.edu/cgi-bin/hgTracks?org=Rat&db=rn4&position=chr10:54304004-54304008) |
| chr10 | 54304074 | 54304078 | NM_053484 | [Gas7](http://genome.ucsc.edu/cgi-bin/hgTracks?org=Rat&db=rn4&position=chr10:54304074-54304078) |
| chr10 | 54307424 | 54307428 | NM_053484 | [Gas7](http://genome.ucsc.edu/cgi-bin/hgTracks?org=Rat&db=rn4&position=chr10:54307424-54307428) |
| chr10 | 54309352 | 54309356 | NM_053484 | [Gas7](http://genome.ucsc.edu/cgi-bin/hgTracks?org=Rat&db=rn4&position=chr10:54309352-54309356) |
| chr10 | 54309372 | 54309376 | NM_053484 | [Gas7](http://genome.ucsc.edu/cgi-bin/hgTracks?org=Rat&db=rn4&position=chr10:54309372-54309376) |
| chr10 | 54309386 | 54309390 | NM_053484 | [Gas7](http://genome.ucsc.edu/cgi-bin/hgTracks?org=Rat&db=rn4&position=chr10:54309386-54309390) |
| chr10 | 54309391 | 54309395 | NM_053484 | [Gas7](http://genome.ucsc.edu/cgi-bin/hgTracks?org=Rat&db=rn4&position=chr10:54309391-54309395) |
| chr10 | 54312435 | 54312439 | NM_053484 | [Gas7](http://genome.ucsc.edu/cgi-bin/hgTracks?org=Rat&db=rn4&position=chr10:54312435-54312439) |
