# Supplementary material for: Identification of direct regulatory targets of the transcription factor Sox10 based on function and conservation
Source: BMC Genomics. 2008 Sep 11;9:408. doi: 10.1186/1471-2164-9-408 (PMC2556353; doi:10.1186/1471-2164-9-408)
Supplement: Additional file 3 — Oligonucleotide primers used for ChIP assay. List and sequence of oligonucleotide primers used for ChIP assay. [file 1471-2164-9-408-S3.doc]

| **primer pair #** | **gene** | **sense** | **anti-sense** |
| --- | --- | --- | --- |
| 1 | **Cmkor1** | GGTGGGGATGGATTAATTCAC | CACAGTTCACTTGGAGAGCAGG |
| 2 | **Cmkor1** | CTAAGCTGGTGGGAATGCAG | TCCCACCAACACATGAGACAG |
| 3 | **Gda** | TGCTGTGAAGAACAGGACACG | ACCTGAAACCTCGCACACAG |
| 4 | **PLP** | TGGCTCTCTTGAGCCTGGTC | TTGGCCTTGACCATGGAATC |
| 5 | **PLP** | CCTGAGTAGGGCTTTGTGCTAAC | TGCAGTTTTCCCCTCCTCC |
| 6 | **PLP** | TTCTGGGAGGAGGGGAAAAC | CCACACAATTCCCAGCTTGTC |
| 7 | **PLP** | GCTGGGGTGAGTGAATGGTC | TTGGACACGGTCCTCCATAAC |
| 8 | **Sox10** | GGTGGATAGTGGGACACAAAGAG | ATTGTCCAAGGCCAGCGAG |
| 9 | **SOD3** | CACACCACAGTCCTGGAGAGAG | AGACGCAGTCAGTGACCTTCAG |
| 10 | **Erbb3** | GCTAGCACACAGCCCCTATTG | GGGAGTGCTGCTAAGGATGG |
| 11 | **Ptn** | TGTTCCTCTGTGCCCCACTG | CTGCCATCACCTGACTCAATTG |
| 12 | **Ptn** | AGCCCTGCTAGTGCCAAATG | CCAACCCTCAACCTGAGAGC |
| 13 | **Ptn** | TTTCCCCTCCCTTCCTTTG | TGCCTGCTCCCATTTTTCC |
| 14 | **Ptn** | GCCTGTATGGCGTCAGTGAG | CTTACAGCGTGGGCGTTATG |
| 15 | **Ngfr** | CTCTCTGCCACTCCCGAATC | TCCCTGGGGAGTTGTAGGAC |
| 16 | **Gzmb** | AAGTCTGCAGAGCCACACCAC | TCAAAAGCGTGGGATGTGTC |
| 17 | **Gas7** | TAAGGCAGGCTCAGGGAGTC | CATGCACGCCTTTCCAGATAC |
|  | **Sod3-control** | CATTGTGTAGCCCTGTGACTGC | TAGAGCTTCTCCTGCACTCCTG |
|  | **β-actin-control** | GGCTACAGCTTCACCACCACA | CAGGAGGGAAGGCTGGAAGAG |
