# Supplementary material for: Identification of direct regulatory targets of the transcription factor Sox10 based on function and conservation
Source: BMC Genomics. 2008 Sep 11;9:408. doi: 10.1186/1471-2164-9-408 (PMC2556353; doi:10.1186/1471-2164-9-408)
Supplement: Additional file 4 — Oligonucleotide primers used for RT-PCR. List and sequence of oligonucleotide primers used for RT-PCRassay. [file 1471-2164-9-408-S4.doc]

| **Gene** | **sense** | **anti-sense** |
| --- | --- | --- |
| **Cmkor1** | GTGGTCTTCCTGGTGTGTTGG | AGAGCACAGGGTTGACACAGC |
| **Gda** | GAAAAGCCTCACCCTCAAAG | ATCAATGGGAGAGTCCGATG |
| **PLP** | ACCACCTGCCAGTCTATTGC | CTCGGCTGTTTTGCAGATG |
| **Sox10** | AGAAAGTTAGCCGACCAGTACC | TGGTACTTGTAGTCCGGATGG |
| **Erbb3** | CAGCGACACAGCCTGCTTAC | TACCCCTTCTCTTCCGGTTC |
| **Ptn** | AATACCAGTTCCAGGCTTGGG | TGATTCCGCTTGAGGCTTG |
| **SOD3** | CGTTCTTGGGAGAGCTTGTC | AGGTCTTTGGAGTGCGTGTC |
| **Ngfr** | CACCGGAGGGAGAGAAACTG | ACCTCCTCACGCTTGGTCAG |
| **Gzmb** | TTCGACAAAGCCAATGAGATATG | GAATGCCCGTGGAGTTGAAC |
| **Gas7** | TCAAGAAGGCACGGAGGAAG | TCATGCCGCAGCTGTGTG |
| **Gapdh** | CAAGAAGGTGGTGAAGCAGG | CCGTATTCATTGTCATACCAGG |
